# Behavioral Outcomes Associated with Hormonal Sterilization in a Questionnaire-Based Study of Cats

**DOI:** 10.3390/vetsci13050428

**Published:** 2026-04-28

**Authors:** Mihaela Velkovska, Maja Zakošek Pipan, Irena Bolko, Darja Pavlin

**Affiliations:** 1 Clinic for Reproduction and Large Animals, Veterinary Faculty, University of Ljubljana, 1000 Ljubljana, Slovenia; mihaela.velkovska@vf.uni-lj.si (M.V.); maja.zakosekpipan@vf.uni-lj.si (M.Z.P.); 2 Faculty of Social Sciences, University of Ljubljana, Kardeljeva pl. 5, 1000 Ljubljana, Slovenia; irena.bolko@fdv.uni-lj.si; 3 Clinic for Small Animals, Veterinary Faculty, University of Ljubljana, 1000 Ljubljana, Slovenia

**Keywords:** cats, deslorelin, flare-up, behavioral changes

## Abstract

Treatment of cats with pharmacological reproductive control is becoming increasingly common, with deslorelin implants as the primary non-surgical method. However, few studies have systematically documented behavioral changes after implantation. This study evaluated 28 behaviors, grouped into eight categories, in 82 cats at one and three months post-deslorelin implantation. At the group level, behavioral changes over time showed a consistent pattern, with decreases in reproductive behaviors, inappropriate elimination, and activity and increases in positive social behaviors and feeding and drinking from one to three months post-implantation. Given the large number of statistical comparisons performed, these findings should be interpreted with caution in the context of multiple testing. Sex-related differences were limited, with females showing a greater increase in feeding and drinking than males at one month. Age-related effects were also observed, with cats younger than one year being more active than those older than three years at one month. Coat type was associated with behavioral variation at three months, with shorthaired cats showing higher levels of reproductive behavior and inappropriate elimination compared with longhaired cats; however, these effects should be interpreted cautiously given multiple testing considerations.

## Simple Summary

Deslorelin implants are the most commonly used method of non-surgical reproductive control in cats. In this study, behavioral changes were assessed one and three months after implantation. By the third month, reproductive behaviors, inappropriate elimination, and activity levels generally decreased, while positive social behaviors and feeding and drinking tended to increase. Females were feeding more frequently than males during the first month. Age and coat type were also associated with behavioral variation: shorthaired cats showed more reproductive behavior, and older cats were less active three months after implantation. These findings support deslorelin’s suppressive effect on reproductive behavior, while also suggesting that age, sex, and coat type are associated with variation in behavioral responses to hormonal suppression.

## 1. Introduction

Uncontrolled breeding of companion animals presents ongoing welfare and public health challenges, contributing to overpopulation, infectious diseases, and animal abandonment [1,2]. The ideal method of reproductive control should minimize adverse effects while ensuring long-term health and behavioral stability [3]. Surgical sterilization remains the most common method for cats and dogs [4], but it is unsuitable for some animals or owners due to factors such as age, breed, health status, lifestyle, and owners’ beliefs or awareness [3]. Concerns about anesthesia, irreversibility, and potential behavioral changes often cause reluctance toward surgical sterilization [5,6]. As a result, there is increasing demand for non-surgical, reversible alternatives. Hormonal sterilization is currently the most promising non-surgical option because of its reversibility and minimal invasiveness [7].

Among hormonal agents, gonadotropin-releasing hormone (GnRH) agonists such as deslorelin (Suprelorin™, Virbac, Carros, France) have gained prominence in recent years. Deslorelin is currently the only GnRH agonist approved in the European Union for use in ferrets, male dogs, prepubertal female dogs, and male cats, while its use in adult female cats remains off-label [8]. It is marketed as a 4.7 or 9.4 mg implant and administered subcutaneously in the intrascapular or periumbilical region [3]. Since its introduction in the early 2000s, deslorelin has been extensively studied in dogs [9,10,11,12,13,14,15] and, more recently, in cats [16,17,18,19,20,21,22,23]. Previous studies in cats have confirmed its ability to suppress reproductive behaviors and aggression [16,18,19,20]; however, behavioral responses have not been systematically evaluated across different demographic variables, such as sex, age, and breed-related traits, nor across clearly defined time points.

GnRH agonists initially overstimulate pituitary GnRH receptors, causing transient increases in luteinizing hormone (LH) and follicle-stimulating hormone (FSH) secretion and a temporary rise in gonadal hormone levels—a period known as the flare-up phase [24]. During this phase, cats may exhibit increased libido, urine marking, vocalization, activity, and appetite [18,25,26,27], and females may remain fertile and able to conceive [17]. This is followed by a downregulation phase, during which desensitization of pituitary GnRH receptors suppresses the release of LH, FSH, and gonadal hormones, resulting in cessation of reproductive behaviors [24,25,26,28,29]. Although pituitary FSH and LH secretion during this phase have not been characterized in cats, suppression appears to involve testicular luteinizing hormone receptor (LHR) downregulation in males [20] and ovarian LHR mRNA downregulation in females [21]. Suppression of gonadal hormone levels has been confirmed in multiple studies [16,18,19,20,22,26,27].

Deslorelin’s duration of action is dose-dependent and highly variable. The 4.7 mg implant typically suppresses fertility for at least 6 months in male dogs and up to 16–37 months in female cats, while the 9.4 mg implant extends efficacy by 1.5 to 2 times [19,22,26,29]. However, the reported duration varies substantially across studies, likely reflecting differences in study conditions (e.g., implant dose, timing relative to the reproductive cycle, and criteria for defining suppression and recovery), as well as population characteristics and inter-individual variability [22,24,26,29,30]. Breed differences in implant duration have also been reported in cats, with British Shorthair, Ragdoll, and Siberian males showing the longest effects [30]. Clinically, this variability is important because it limits precise prediction of the duration of fertility suppression in individual animals and should therefore be considered when planning breeding management and follow-up after treatment. Importantly, the effects of deslorelin are reversible, with normal reproductive function resuming after implant removal, although the interval to recovery may also vary among individuals [18,19,25,26].

Cats have rarely been studied for behavioral responses to deslorelin implantation [8,18,20,25,27], typically only as secondary outcomes. As a result, behavioral data are often collected without standardized or validated measures and are not the primary focus of study design, which limits their interpretability and clinical applicability. Previous research directly comparing behavior in cats treated with deslorelin implants and those undergoing surgical gonadectomy showed that both interventions resulted in similar reductions in sexually dimorphic reproductive behaviors, while general temperament and social interactions remained largely stable over time [31]. These findings support the use of deslorelin implantation as a viable and reversible alternative to surgical sterilization regarding its behavioral effects.

In cats, reproductive behavior is linked to a range of hormonally driven physiological and behavioral changes. In females, estrus is typically marked by increased vocalization, restlessness, rolling, lordotic posture, and more affiliative or attention-seeking behavior. Males commonly display roaming, urine marking, mounting attempts, and increased aggression [17,25,26,27]. Many of these behaviors are considered problematic by owners, especially persistent vocalization, urine marking, and increased activity or escape attempts, as they can disrupt the household environment and complicate the management of intact animals. Therefore, behavioral changes associated with reproductive status are an important clinical consideration when evaluating the effectiveness of reproductive control methods.

This study was designed to evaluate behavioral changes as the primary objective using a questionnaire-based approach. Specifically, behavioral patterns were compared across two distinct physiological phases: flare-up and downregulation. In addition, associations between behavioral changes and factors such as sex, breed, and age were analyzed, along with potential correlations among individual behavioral categories.

## 2. Materials and Methods

### 2.1. Data Collection

This cross-sectional study analyzed data from anonymous online questionnaires voluntarily completed by cat owners worldwide. Cats were eligible if they had received at least one deslorelin implant and at least three months had passed since the last implant. This interval was defined to ensure that cats had progressed beyond the initial flare-up phase and had entered a more stable downregulation phase, allowing owners to report behavioral changes across both phases within the scope of the questionnaire. There were no breed restrictions. The questionnaire link was distributed through a study-specific email to four FIFe-affiliated European feline federations—the Slovenian Feline Federation, Felis Belgica (Belgium), Sveriges Kattklubbars Riksförbund (Sweden), and Felis Britannica (UK), which forwarded the invitation to their members by email. Links were also posted in Facebook (Meta Platforms, Inc., Menlo Park, CA, USA) cat breeder groups and promoted with QR-coded flyers at international cat shows.

The questionnaire was hosted on the GDPR-compliant (Regulation [EU] 2016/679, General Data Protection Regulation. European Parliament and Council: Brussels, Belgium, 2016.) 1KA platform, and participation was anonymous. Respondents were informed in advance that they could discontinue at any time. Informed consent was obtained through completion and submission of the questionnaire, as stated in the introductory text, which specified that data would be used for research purposes.

This study was approved by the University of Ljubljana Committee for Ethics in Research Involving Human Subjects (approval no. 052-2025).

### 2.2. Questionnaire

The questionnaire was available in Slovenian and English and consisted of three sections designed to collect information on the cats’ characteristics, reproductive history, and behavioral and clinical changes following deslorelin implantation.

Section 1—General information

This section collected demographic and environmental data, including sex, age, breed, living environment (indoor, outdoor, mixed), primary purpose of ownership (pet or breeding), and the presence of other cats in the household.

Section 2—Reproductive history and implant use

This section covered the animals’ reproductive history (number of litters and kittens) and details related to deslorelin treatment: number, timing, and intervals of deslorelin implantations, post-implant matings/parturitions/litters, and household changes during implant use (relocation, newborns).

Section 3—Behavioral assessment

This section assessed 28 distinct behavioral and clinical changes (Table 1) using a 5-point Likert scale (1 = much less, 2 = less, 3 = same, 4 = more, 5 = much more) at 1 month (flare-up) and 3 months (downregulation) post-implantation. Owners rated perceived changes in the frequency or intensity of each behavior between these two time points. The options “do not remember” and “not applicable” were provided for behaviors never observed or when less than three months elapsed since implantation. An open-ended comments section concluded the survey.

### 2.3. Statistical Analysis

**Figure 1:**
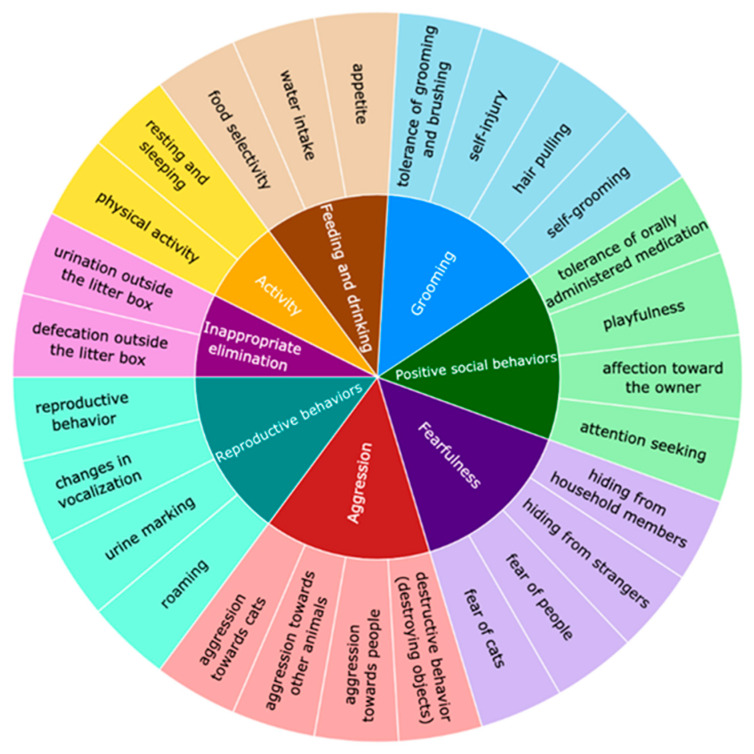
Clustering of 28 individual behavioral variables into eight ethologically similar groups, based on conceptual and presumed biological similarity. Subsections represent individual behaviors within each group.

Data were exported to Microsoft Excel^®^ and categorized as complete or partial based on the proportion of items answered. Only fully completed or partially completed questionnaires meeting a predefined minimum response threshold (≥65% of items answered) were included in the analysis. All statistical analyses were conducted using IBM SPSS Statistics (version 27.0). Cats were classified into three age groups: group 1 (<1 year), group 2 (1–3 years), and group 3 (>3 years). Breed distribution was additionally reported descriptively at the level of individual breeds, whereas for statistical analyses breeds were categorized as shorthaired or longhaired due to small subgroup sizes. Descriptive statistics–including frequencies, proportions, means ± standard deviations (SD), and medians (IQR)—were used to characterize the study population, reproductive history, and behavioral responses. To facilitate descriptive interpretation, responses on the five-point Likert scale were recoded into three categories: less (original scores 1–2), same (score 3), and more (scores 4–5). This recording was applied exclusively for descriptive presentation of directional changes. All inferential statistical analyses were performed on the original five-point Likert scale data. Responses marked “do not remember” or “not applicable” were treated as missing data. The 27 behavioral variables were clustered into eight ethologically similar groups based on conceptual and presumed biological similarity (Figure 1). The variable male cat urine odor intensity was analyzed separately, as it applied only to male cats.

To ensure directional consistency within groups, semantically opposite behaviors were reverse-coded as follows: resting and sleeping within the *Activity* group, food selectiveness within the *Feeding and Drinking* group, and hair pulling and self-injury within the *Grooming* group. Behavioral groups are presented in italics with initial capitalization to distinguish them from individual behavioral variables, which are written in lowercase.

Associations between behavioral groups at one and three months post-implantation were calculated using Kendall’s τ (|τ|: 0.00–0.30 negligible; 0.30–0.50 low; 0.50–0.70 moderate; 0.70–0.90 high; 0.90–1.00 very high) [32]. Changes in individual behaviors and behavioral categories were compared at one and three months post-implantation using the Wilcoxon signed-rank test. Differences between sexes and between shorthaired and longhaired cats were assessed using the Mann–Whitney U test for independent samples. Differences among age groups were analyzed using the Kruskal–Wallis test for independent samples. Behavioral analyses were conducted at two levels. Primary analyses focused on aggregated behavioral groups to reduce the number of statistical comparisons and in-crease interpretability. Analyses of individual behaviors were considered exploratory. To control for inflation of Type I error due to multiple comparisons, the Benjamini–Hochberg false discovery rate (FDR) correction was applied separately to predefined families of related tests (behavioral groups, individual behaviors, and correlation analyses). Where applicable, post hoc pairwise comparisons following Kruskal–Wallis tests were performed using Bonferroni adjustment. Both unadjusted and FDR-adjusted significance were considered when interpreting results, with statistical offset at α = 0.05 after correction.

## 3. Results

### 3.1. General Characteristics of the Sample

A total of 97 questionnaires were submitted; 74 (76.3%) were fully completed, and 23 (23.7%) were partially completed. After excluding 15 questionnaires that did not meet the predefined minimum response threshold of 65% of items answered, the final analytical sample included 82 questionnaires. Thirty questionnaires (36.6%) were completed in Slovenian, and 52 (63.4%) in English. The mean age of the cats at the time of implantation was 29.5 ± 19.6 months (range: 6–96 months). Shorthair breeds included Abyssinian, Asian, Bengal, British Shorthair, Burmese, Chartreux, Cornish Rex, Domestic European, Don Sphynx, Oriental, Peterbald, Pixie-bob, Russian Blue, and Sphynx. Longhair breeds included American Bobtail, Birman, Maine Coon, Neva Masquerade, Norwegian Forest, LaPerm, Ragdoll, Siberian, Somali, and Turkish Van. The most represented breed was the Maine Coon, accounting for 22% of the study population. All owners reported that at least one other cat lived in the same household as the cat described in the questionnaire. Descriptive statistics for the sample characteristics are presented in Table 2.

### 3.2. Reproductive History and Deslorelin Use

Thirty-three cats (40%) received the deslorelin implant more than once. Of these, 12 owners (36.4%) reported a specific time interval between implantations, nine owners (27.3%) reported reimplantation after a litter, and two owners (6.1%) indicated reimplantation when undesired behavioral changes occurred. Only nine cats (27.3%) received a new implant before the onset of fertility in males or estrus in females.

Among the 30 female cats in the study, 19 (63.3%) had given birth before implantation, while 11 (36.7%) had not. After implant use, 42 cats (51.2%) did not experience successful mating or estrus, while 40 cats (48.8%) did. Eight females (26.7%) had litters both before and after implantation. In the subgroup of eight females that had litters both before and after implantation, the mean total number of kittens per cat decreased from 4.8 before implantation to 3.7 after implantation.

Seven owners (8.5%) reported household changes during the implant use period that could have affected their cats’ behavior. Of these, five owners (71.4%) reported relocation, and two owners (28.6%) reported the arrival of a new animal in the household.

### 3.3. Behavioral Changes

#### 3.3.1. Relevance and Stability of Behaviors

A substantial proportion of owners reported that several behaviors were either non-relevant or unchanged after implantation. Detailed results are presented in Table 3.

#### 3.3.2. Behavioral Groups: Overall Changes and Correlations

One month after implantation, owners reported decreased expression of *Reproductive behaviors* in 55.8% of cats, *Inappropriate elimination* in 30.8%, *Aggression* in 32.9%, *Fearfulness* in 21.8%, and *Activity* in 16.4%. In contrast, an increased expression was reported for *Positive social behaviors* in 19.6% of cats and *Feeding and drinking* in 16.5% of cats. These results are based on recoded descriptive categories.

**Figure 2:**
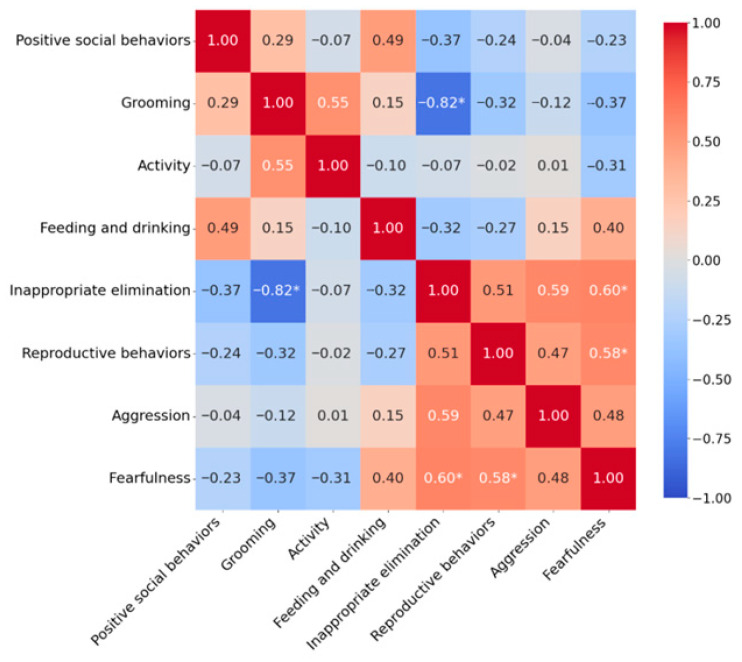
Correlations between behavioral groups one month after deslorelin implant insertion. Correlation coefficients were calculated using Kendall’s tau rank correlation test. Statistically significant correlations are marked with an asterisk at *p* < 0.05.

At one month, a significant strong negative correlation was observed between *Inappropriate elimination* and *Grooming* (τ = −0.816, *p* = 0.015) (Figure 2). Significant moderate positive correlations were found between *Inappropriate elimination* and *Fearfulness* (τ = 0.604, *p* = 0.021) and between *Reproductive behaviors* and *Fearfulness* (τ = 0.581, *p* = 0.023). However, after applying the Benjamini–Hochberg FDR correction for multiple comparisons, none of these associations remained statistically significant.

**Figure 3:**
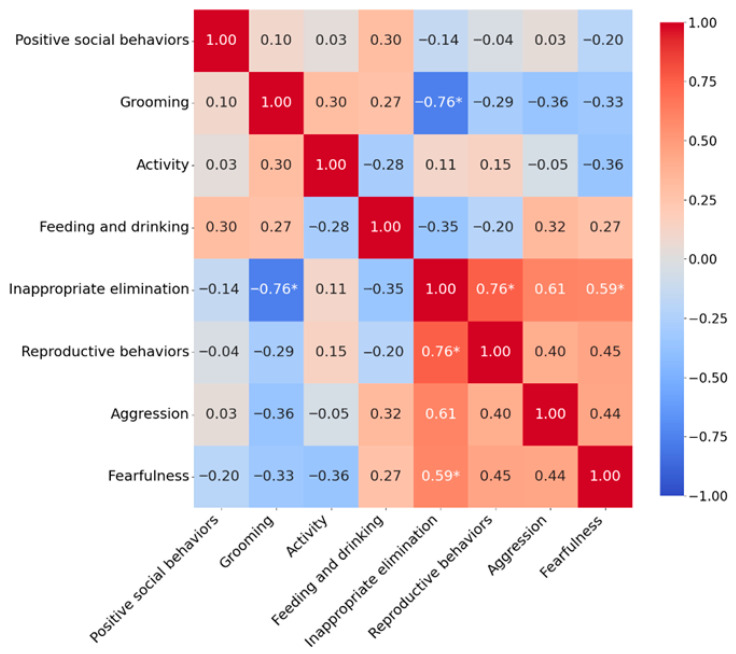
Correlations between behavioral groups three months after deslorelin implant insertion. Correlation coefficients were calculated using Kendall’s tau rank correlation test. Statistically significant correlations are marked with an asterisk (*p* < 0.05).

Three months after implantation, the previously mentioned changes were reported in a higher proportion of cats. A decreased expression was reported for *Reproductive behaviors* in 77.1% of cats, *Inappropriate elimination* in 57.5%, *Activity* in 29.8%, and *Aggression* in 41.2%. In contrast, an increased expression was reported for *Positive social behaviors* in 36.8% of cats and *Feeding and drinking* in 28.4% of cats. At this time point, a statistically significant strong negative correlation was again observed between *Inappropriate elimination* and *Grooming* (τ = −0.759, *p* = 0.039), as well as a significant strong positive correlation between *Inappropriate elimination* and *Reproductive behaviors* (τ = 0.756, *p* = 0.034), and a moderate positive correlation between *Inappropriate elimination* and *Fearfulness* (τ = 0.587, *p* = 0.036) (Figure 3). Similar to the findings at one month, these correlations did not remain statistically significant after FDR correction.

#### 3.3.3. Changes in Individual Behaviors Between One and Three Months

Comparison of behavioral groups between one and three months after implantation showed that *Positive social behaviors* (W = 154.5, Z = 3.02, *p* = 0.003) and *Feeding and drinking* (W = 171.0, Z = 3.79, *p* < 0.001) were significantly more expressed after three months, while *Activity* (W = 0, Z = −3.68, *p* < 0.001), and *Reproductive behaviors* (W = 0, Z = −2.70, *p* = 0.007) decreased significantly over time. These changes remained statistically significant after FDR and correction. Although *Inappropriate elimination* showed a decrease over time (W = 0, Z = −2.07, *p* = 0.038), this change did not remain statistically significant after correction.

Statistically significant differences between the first and third months were also observed in several individual behaviors. Behaviors that occurred less frequently three months after deslorelin implantation compared to one month after implantation included reproductive behavior (W = 43.0, Z = −5.00, *p* < 0.001), changes in vocalization (W = 10.5, Z = −4.51, *p* < 0.001), urination outside the litter box (W = 0, Z = −3.74, *p* = 0.001), urine marking (W = 0, Z = −4.20, *p* < 0.001), and male cat urine odor intensity (W = 0, Z = −4.28, *p* < 0.001). These changes remained statistically significant after FDR correction. Conversely, the following behaviors occurred more frequently at three months after implantation than at one month: resting and sleeping (W = 153.0, Z = 3.82, *p* = 0.001), attention seeking (W = 171.0, Z = 3.14, *p* = 0.002), affection toward the owner (W = 283.0, Z = 3.43, *p* < 0.001), and appetite (W = 300.0, Z = 4.52, *p* < 0.001). These changes also remained significant after correction. Other observed differences, including decrease in aggression towards cats (W = 54, Z = −2.49, *p* = 0.013), aggression towards other animals (W = 9, Z = −2.00, *p* = 0.046), physical activity (W = 19.5, Z = −2.24, *p* = 0.025), and food selectivity (W = 0, Z = −2.27, *p* = 0.023), as well as increase in playfulness (W = 116.5, Z = 2.61, *p* = 0.009) and water intake (W = 15.0, Z = 2.24, *p* < 0.025) did not remain statistically significant after correction for multiple comparisons.

#### 3.3.4. Differences by Sex, Age, and Coat Length

One month after deslorelin implantation, females showed a statistically significant greater increase in the *Feeding and drinking* behavioral group than males (U = 196.5, *p* = 0.035). However, this difference did not remain statistically significant after applying the FDR correction for multiple comparisons. No other significant sex-related differences were detected at either time point.

The *Activity* behavioral group one month after implantation differed significantly by age group (H(2) = 6.816, *p* = 0.033), with younger cats appearing more active than older cats. However, this change did not remain statistically significant after FDR correction. Accordingly, post hoc comparisons were not interpreted. No other statistically significant age-related differences were observed.

Shorthaired cats showed statistically significantly higher scores for *Reproductive behaviors* (U = 3, *p* = 0.042) and *Inappropriate elimination* (U = 3.5, *p* = 0.018) three months after implantation compared with longhaired cats. However, these differences did not remain statistically significant after FDR correction. No other statistically significant coat length-related differences were observed.

## 4. Discussion

The aim of this study was to compare behavioral changes during the flare-up phase at one month and the downregulation phase at three months after deslorelin implantation. Compared to the flare-up phase, the downregulation phase was associated with reduced reproductive and elimination behaviors, along with a concurrent increase in affiliative and positive social behaviors.

Contrary to expectations, behavioral patterns typically associated with the flare-up phase [8,31] were not clearly detected based on owner-reported questionnaire data. Instead, owners most frequently reported decreases in *Reproductive behavior*, *Aggression*, *Fearfulness*, *Activity* and *Inappropriate elimination*. Concurrently, *Positive social behaviors* and *Feeding and drinking* were reported as either stable or increased. One possible explanation is that endocrine activation during the flare-up phase may not necessarily result in overt behavioral manifestations, with hormonal changes occurring without clear behavioral expression. A previous longitudinal study measuring fecal estradiol concentrations in queens demonstrated hormone concentrations consistent with estrus; although only a minority of animals showed observable behavioral signs [27]. This suggests that subclinical hormonal fluctuations may occur without clear behavioral expression.

Another explanation may be the timing of implantation relative to the reproductive cycle. Previous studies indicate that implantation during diestrus may attenuate reproductive activation, whereas implantation during interestrus or anestrus is more often followed by estrus induction [17,26]. For this reason, measuring serum progesterone concentrations before implantation or inserting the implant during diestrus is recommended to reduce side effects [17]. Adherence to such clinical recommendations may have reduced the likelihood of observable flare-up behaviors in the present study population. In addition, recall bias inherent in owner-reported questionnaires may have limited the detection of transient or subtle behavioral changes occurring during the early post-implantation period.

The behavioral changes during the flare-up phase may be explained by the underlying endocrine mechanisms associated with GnRH agonist action. Initial stimulation of the hypothalamic–pituitary–gonadal axis causes a transient increase in gonadal hormone concentrations, which may influence sexually dimorphic behaviors. This is followed by pituitary receptor desensitization and suppression of LH and FSH secretion, ultimately leading to reduced production of sex steroids such as estrogen and testosterone [24,25,26]. Because these hormones play a key role in regulating reproductive behavior, aggression, and activity levels, their suppression may contribute to the observed reductions in these behavioral domains. However, given the observational nature of the present study, these mechanisms should be interpreted as plausible explanations rather than direct evidence of causality.

Consistent with these mechanisms, behavioral changes observed during the downregulation phase were more aligned with the expected pharmacodynamic effects of deslorelin. *Reproductive behaviors*, *Aggression*, *Activity*, and *Inappropriate elimination* declined further, while *Positive social behaviors* and *Feeding and drinking* increased, consistent with suppression of gonadal hormone production [24]. These changes are in line with the expected pituitary downregulation and subsequent suppression of gonadal hormone production described in the literature [24], although a direct causal relationship cannot be established within the observational design of the present study. Shorthaired cats showed higher scores for *Reproductive behaviors* and *Inappropriate elimination* than longhaired cats during the downregulation phase; however, these differences did not remain significant after correction for multiple comparisons and should be interpreted cautiously. Although coat length is unlikely to be causative, breed-related differences in reproductive traits have been described in pedigree cats [33,34], suggesting that phenotypic characteristics may reflect underlying biological variation.

Comparison between phases confirmed an increase in *Positive social behaviors* and *Feeding and drinking*, alongside a decrease in *Reproductive behaviors* and *Activity*. These changes reflect the expected transition from the flare-up to the downregulation phase [24].

Age- and sex-related behavioral differences were most evident during the flare-up phase but did not remain statistically significant after correction for multiple comparisons. Younger cats (<1 year) appeared more active than older cats (>3 years) during flare-up, and females showed a greater increase in *Feeding and drinking*. Increased appetite may be consistent with documented metabolic effects of gonadal suppression [18], and previous studies have shown that weight gain after sterilization is primarily associated with increased energy intake [35], with females more likely than males to exhibit a decline in body condition [36]. However, data on body weight were not collected, which may have influenced the interpretation of feeding and drinking behaviors.

A moderate positive correlation between *Fearfulness* and *Inappropriate elimination* during the first month suggests an association between these behavioral domains, possibly reflecting shared underlying factors such as emotional reactivity. A strong negative correlation between *Inappropriate elimination* and *Grooming* during this period further indicates that these behaviors tend to occur in opposite directions within individuals, potentially reflecting differences in stress-related behavioral patterns. In the third month, a strong positive correlation between *Reproductive behaviors* and *Inappropriate elimination* was observed, indicating that these behaviors may vary in parallel within the same individuals. The persistence of a moderate positive correlation between *Fearfulness* and *Inappropriate elimination* at this stage suggests that emotionally reactive cats may be more likely to exhibit elimination-related behaviors. A study on separation anxiety in cats reported that inappropriate elimination is a frequent symptom of this behavioral disorder [37]. Overall, these findings indicate associations between emotional and elimination-related behaviors, which may be influenced by endocrine, environmental, and individual factors. However, none of these correlations remained statistically significant after correction for multiple comparisons and should therefore be interpreted as exploratory.

A decrease in mean litter size was observed after deslorelin implantation, but this finding should be interpreted cautiously. Fertility is influenced by multiple factors, including age-related decline in older females [34], natural variability in reproductive capacity between breeds [33,34], and potentially reduced ovarian activity shortly after deslorelin implantation [38]. Environmental factors such as stress, nutrition, and seasonal fluctuations should also be considered, as they may affect mating success and litter size.

One of the main limitations of this study is the lack of baseline behavioral data before deslorelin use. This prevents definitive confirmation that the observed changes were directly caused by the implant; instead, they can only be associated with the time elapsed since implantation. Another limitation is that the questionnaire did not include information on implant dosage (4.7 or 9.4 mg), which prevents evaluation of potential dose-dependent effects on behavior. To date, no evidence suggests that deslorelin dosage affects behavior, although it does influence the duration of reproductive suppression [26,28,29].

The composition of the study population represents an additional limitation. The sample consisted predominantly of purebred or pedigreed cats, which may have been kept in breeding environments. Such cats can be managed under different housing, social, and reproductive conditions than the general pet cat population, which largely consists of non-pedigreed companion animals. These factors may influence baseline behavior and responses to hormonal treatment. Furthermore, hormonal sterilization with deslorelin is more commonly used in pedigree cats intended for breeding than in the general pet population. Consequently, the findings may be primarily applicable to breeding populations, and their generalization to the overall pet cat population should be interpreted with caution.

An additional limitation relates to the categorization of breeds into shorthaired and longhaired groups. Although coat length is a readily identifiable and consistently reported trait, it reflects variation in a single genetic characteristic and does not correspond to broader phylogenetic relationships among breeds. Accordingly, this categorization should be interpreted as a grouping applied for analytical purposes rather than a biologically comprehensive classification.

Behavioral assessment was based on subjective owner evaluations rather than direct observation. This approach, typical of questionnaire-based studies, is susceptible to perceptual bias influenced by owners’ experience and knowledge. These inherent limitations must be considered when interpreting the results. Reporting bias is also possible, as owners may have rated positive changes more favorably and attributed them to deslorelin use. In addition, the exact timing of implantation was unknown, which prevented accurate determination of the time interval between implantation and questionnaire completion.

## 5. Conclusions

Deslorelin effectively suppresses hormonally driven behaviors during the downregulation phase and does not consistently trigger reproductive behavior during the flare-up phase. During the downregulation phase, there was a clear reduction in undesirable behaviors and an increase in positive social behaviors, with the most robust changes observed at the level of aggregated behavioral groups. Evidence for differences related to sex, age, and coat type was not robust after correction for multiple comparisons and should therefore be interpreted with caution. These factors may still contribute to individual variability in response to deslorelin and warrant further investigation in larger and more controlled studies.

## Figures and Tables

**Table 1:** Behavioral variables (*n* = 28), encompassing undesirable, affiliative, and physiological behaviors, assessed using a 5-point Likert scale (1 = much less, 2 = less, 3 = same, 4 = more, 5 = much more) at one month (flare-up phase) and three months (downregulation phase) after deslorelin implantation.

No.	Behavioral/Clinical Change	Description
*Undesirable behaviors*		
1	reproductive behavior	lordotic positioning and acceptance of mounting in females; courtship behavior, and mounting attempts in males
2	aggression towards cats	hissing, growling, swatting, chasing, fighting felines
3	aggression towards other animals	hissing, growling, swatting, chasing, fighting non-felines
4	aggression towards people	hissing, growling, swatting, chasing, biting humans
5	destructive behavior	inappropriate scratching, biting, or chewing objects
6	fear of cats	avoiding interaction with other felines, crouching, hiding, or fleeing
7	fear of people	avoiding interaction with other humans, crouching, hiding, or fleeing
8	changes in vocalization	change of frequency of meowing or yowling
9	hiding from strangers	avoiding interaction with unknown humans, hiding, or fleeing
10	hiding from household members	avoiding interaction, hiding, or fleeing from non-owner household members
11	roaming	escape attempts from home range seeking opposite sex conspecifics
12	hair pulling	excessive hair pulling leading to alopecia
13	self-injury	excessive licking, scratching, biting, or chewing fur, tail mutilation
14	urination outside the litter box	squatting micturition on horizontal surfaces
15	defecation outside the litter box	squatting defecation on horizontal surfaces
16	urine marking	vertical urine spraying with tail quivering, elevated hindquarters
17	male cat urine odor intensity	perceived pungency of urine in male cats
*Affiliative behaviors*		
18	attention seeking	approaching, following, allorubbing, purring toward humans
19	affection towards the owner	approaching, following, allorubbing, purring towards the owner
20	playfulness with people	play-fighting, object play, running, and jumping
*Physiological behaviors*		
21	resting and sleeping	recumbent rest and sleep duration and/or frequency
22	physical activity	jumping, climbing, running frequency
23	appetite	feeding motivation and intake volume
24	water intake	drinking frequency and intake volume
25	food selectivity	refusal of familiar foods
26	self-grooming	licking, scratching, biting, or chewing fur
27	tolerance of grooming and brushing	compliance during coat brushing
28	tolerance of orally administered medication	compliance during peroral pharmacotherapy

**Table 2:** Sample characteristics of the study population (*n* = 82).

Individual Characteristic	Category	Number (%)
Gender	Male	52 (63.4)
	Female	30 (36.6)
Age	<1 year	8 (9.8)
	1–3 years	53 (64.6)
	>3 years	21 (25.6)
Breed	Shorthair	30 (36.6)
	Longhair	52 (63.4)
Living environment	Indoor	72 (87.8)
	Outdoor	2 (2.4)
	Indoor and outdoor	8 (9.8)
Purpose	For breeding	71 (86.6)
	As pets	7 (8.5)
	Other	4 (4.9)
Total		82 (100)

**Table 3:** Behaviors reported as non-relevant or unchanged (score 3, “same”) by more than 50% of owners at one and three months after deslorelin implantation.

Behavior Category	Behavior	Non-Relevant (1 and 3 Months)	Unchanged (1 Month)	Unchanged (3 Months)
*Aggression*	aggression towards cats			
	aggression towards other animals	x		
	aggression towards people	x		
	destructive behavior	x		
*Fearfulness*	fear of cats	x		
	fear of people	x		
	hiding from strangers	x		
	hiding from household members	x		
*Reproductive behaviors*	reproductive behavior			
	roaming	x		
	urine marking	x*		
	changes in vocalization			
*Positive social behaviors*	playfulness		x	x
	attention seeking		x	
	affection towards the owner		x	
	tolerance of orally administered medication		x	x
*Grooming*	tolerance of grooming and brushing		x	x
	self-grooming		x	x
	hair pulling	x		
	self-injury	x		
*Feeding and drinking*	appetite		x	
	water intake		x	x
	food selectivity		x	x
*Activity*	physical activity		x	x
	resting and sleeping		x	
*Inappropriate elimination*	urination outside the litter box	x		
	defecation outside the litter box	x		

x indicates behaviors meeting this criterion. x* indicates urine marking reported as non-relevant by more than 50% of owners three months after implantation.

## Data Availability

The raw data supporting the conclusions of this article will be made available by the authors on request.

## Abbreviations

The following abbreviations are used in this manuscript:

GnRH	Gonadotropin-releasing hormone
FSH	Follicle-stimulating hormone
LH	Luteinizing hormone
LHR	Luteinizing hormone receptor
mRNA	Messenger ribonucleic acid
GDPR	General Data Protection Regulation
